# State of the art of rare disease activities in Europe: a EUCERD perspective

**DOI:** 10.1186/1750-1172-7-S2-A1

**Published:** 2012-11-22

**Authors:** Ségolène Aymé

**Affiliations:** 1 Inserm, US14, Orphanet, Paris, France

## 

**Figure 1:**
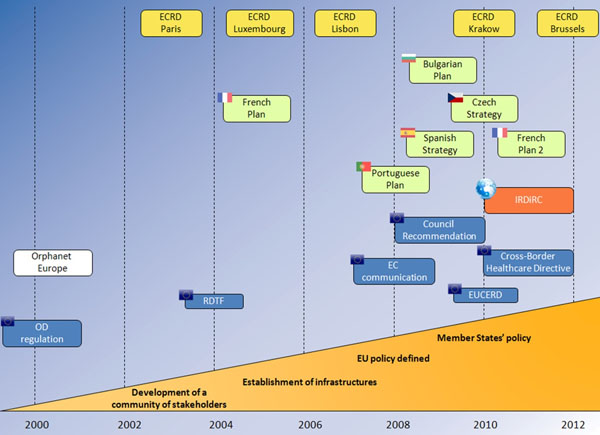
Emergence of concepts and initiatives surrounding rare diseases in Europe

**Figure 2:**
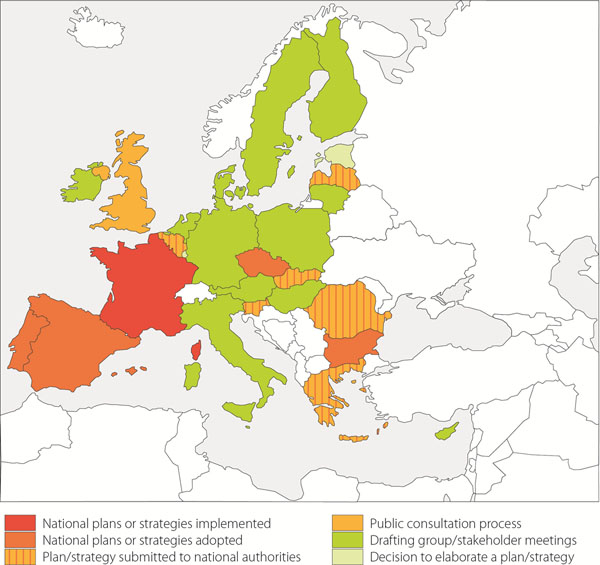
Current stages of development of national plans or strategies for rare diseases in EU MS (in December 2011).

**Figure 3:**
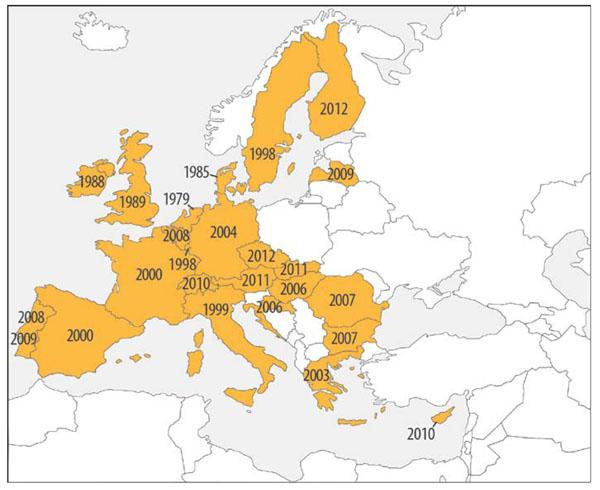
Countries in Europe with a national alliance for rare disease patient organisations and year founded

Two years ago, at the ECRD in Krakow, we were celebrating the recommendation of the Council of Ministers on an action in the field of rare diseases: this recommendation requests that Member States define and implement a plan or a strategy for rare diseases by the end of 2013 and asks the European Commission to establish a EU Committee of Experts in the field of Rare Diseases (EUCERD) as a forum of stakeholders to propose action points at EU level. Two years on, there is enough evidence to judge the outcome of Recommendation which can be qualified as positive despite the economic crisis. The dynamic of action is still impressive (Figure 1). The EUCERD has been established and fulfills its mission as expected. The publication of the EUCERD annual report on the state of art of rare disease activities in Europe testifies the many achievements [1]. The EUCERD adopted a set of quality criteria for the designation of centres of expertise for rare diseases at national level. This work served as a basis for the working group on centres of expertise in the framework of the Cross Border Healthcare Directive. All countries are now engaged in the process of elaborating a plan or a strategy for rare diseases (Figure 2). The recent developments in genomics now translate into more diagnostic tests for rare diseases. So far over 1,800 rare diseases can be tested in one EU country. Targeted funding for rare diseases has also produced its effects, with more transnational cooperation which translated into the important decision to establish an International Consortium to fund research: the IRDiRC. The Consortium will allow more ambitious goals to be set and achieved faster and it will ease the mobilisation of the critical mass of expertise and resources whilst avoiding overlaps in research. The Regulation on Orphan Medicinal Products is still producing positive effects with currently over 70 products with a marketing authorisation in the EU and many more in development. The Orphanet database became a Joint Action between all Member States, showing the great degree of willingness to provide unified, high-quality information to all EU citizens. Last but not least, patient organisations are increasingly better organised to make their voice heard. Not only is EURORDIS the voice of patients in Europe, but umbrella organisations have been established in most countries (Figure 3).
